# The Link Between Cannabis Use and Violent Behavior in the Early Phase of Psychosis: The Potential Role of Impulsivity

**DOI:** 10.3389/fpsyt.2022.746287

**Published:** 2022-03-22

**Authors:** Valerie Moulin, David Framorando, Jacques Gasser, Elise Dan-Glauser

**Affiliations:** ^1^Unit for Research in Legal Psychiatry and Psychology, Institute of Forensic Psychiatry, Department of Psychiatry, Lausanne University Hospital, Lausanne, Switzerland; ^2^Institute of Psychology, University of Lausanne, Lausanne, Switzerland

**Keywords:** cannabis use, impulsivity, violent behavior, psychosis, prevention

## Abstract

**Introduction:**

Recently, the literature has shown that Cannabis Use (CU) was a risk factor for Violent Behavior (VB) in patients with psychosis, and those in the early phase of psychosis (EPP). These findings are relevant because of the high prevalence of CU in this EPP, and the potential for prevention during this phase of illness. However, there is still a lack of clear explanations, supported by empirical evidence, about what underlies the link between CU and VB against other.

**Method:**

This viewpoint reviews the scientific literature on the link between CU and VB, and the involvement of impulsivity in this relationship. This last point will be addressed at clinical and neurobiological levels.

**Results:**

Recent studies confirmed that CU is particularly high in the EPP, and is a risk factor for VB in the EPP and schizophrenia. Studies have also shown that impulsivity is a risk factor for VB in psychosis, is associated with CU, and may mediate the link between CU and VB. Research suggests a neurobiological mechanism, as CU affects the structures and function of frontal areas, known to play a role in impulsive behavior.

**Conclusion:**

Scientific evidence support the hypothesis of an involvement of impulsivity as a variable that could mediate the link between CU and aggression, particularly, when CU has an early onset. However, this hypothesis should be confirmed with longitudinal studies and by taking into account confounding factors. The studies highlight the relevance of early prevention in the EPP, in addition to interventions focusing on psychotic disorders.

## Introduction

A significant number of studies have shown that, compared to the general population, people suffering from psychosis (1–5), and particularly those in the Early Phase of Psychosis (EPP) (6–10), show a high prevalence of violent behaviors (VB) against others. This association is robust, observed in various countries (11, 12).

Patients in EPP are known to be at high risk for VB, as compared to those in later phases of the illness (9, 13). Various early intervention programs have been developed in recent years for EPP (14–18), leading to better psychopathological and social outcomes. The benefits of these programs in reducing VB seem however limited [as highlighted by longitudinal studies (19–21)], probably due to the predominant focus on psychopathological and functional levels, leaving aside VB prevention (9, 19, 22, 23). In addition to the treatment of psychotic disorders in early intervention programs, the prevention of these behaviors needs to be addressed to reduce harmful effects, both in victims and patients themselves. This would reduce their stigmatization, long-term detention in prisons unsuited to their pathology, and difficulties regarding reintegration into society.

This prevention should be based on assessments and interventions that target dynamic risk factors for VB. Dynamic risk factors are those that are likely to change during treatment, as compared to “static” factors, which cannot be modified by treatment (24). Research has identified positive symptoms, substance use, impulsivity, comorbid antisocial personality disorder, lack of insight, hostility, anger, and emotional instability (25–37) as potential dynamic risk factors for VB. Recently, various results have shown that cannabis use (38–40) (CU) could also play a role in the risk of VB. This finding is particularly relevant due to the high prevalence of CU in EPP and the potential for prevention during this phase of the disease.

However, there is still a lack of empirical evidence to explain what underlies the link between CU and VB. Data from the literature and our own research led us to explore impulsivity as a mediating variable that might underlie this link. In this viewpoint, we present the main evidence available in the literature on the links between CU and VB against others, and then discuss the possible involvement of impulsivity in this relationship. This latter point will be addressed at both the clinical and neurobiological levels. We will conclude this article by discussing the value of early prevention of VB in the EPP.

## Cannabis Use and Violent Behavior in the Early Phase of Psychosis

Until the last few years, there has been a lack of interest regarding the effects of CU on VB. This lack of attention may be related to the fact that CU was perceived as a means of enhancing positive emotions, or calming users, rather than stimulating them (41). The study of the impact of CU on VB is very recent (38–40), and probably related to cannabis legalization procedures in different countries, the increase in THC levels, or in relation to the first research specifically showing relationship between cannabis and VB (42, 43).

The use of various substances (without distinction) is known to be a key dimension in the risk for VB and recidivism (28, 44), particularly in people suffering from psychosis. Cannabis is the most widely used recreational drug in the world among young people (18–25 years) (43) and is particularly prevalent among young people with psychotic disorders (45, 46), and mainly in the EPP. In the latter group, high rates of CU (29–38%) are evidenced, which can have both clinical and neurobiological deleterious consequences. Moreover, in subgroups of violent patients with psychosis, our literature review shows elevated levels of CU: ranging from 40 to 49% in chronic patients (47–49), 44 to 64% in EPP (20, 50–52), and reaching a rate of 70% in a sample of 2,102 prisoners at high risk for developing psychosis (53).

The results of two recent meta-analyses (38, 54) on adolescents and young adults on the one hand, and people with severe mental illnesses on the other hand, have shown significant results regarding the link between CU and violence. Among young people (54), a moderate association was found between CU and VB with an Odd Ratio (OR) of 2.11 (including OR of 2.15 for cross sectional studies and 2.02 for longitudinal studies). The results remained significant after controlling for important confounding factors. This study has showed that early and persistent cannabis users experience more mental health problems and delinquency than others.

Results from a second meta-analysis (38) suggest that CU and cannabis misuse significantly increase VB among people with severe mental illnesses (OR of 2 for CU; 5.8 for cannabis misuse, 4.7 for mixed use, and 2.82 for studies that have adjusted for confounding factors). Specifically, in chronic patients with psychosis (40, 55), CU has been shown to be associated with violence, even after controlling for confounding factors such as other substance use, impulsivity, psychopathic traits, personality disorders (39), affective symptoms, and verbal IQ (56). For example, in a birth cohort study, schizophrenic patients with CU have been found to be 3.8 times more likely to be violent than control subjects (3). A recent longitudinal study (57) of 965 patients showed that persistent CU induces subsequent VB, after controlling for stimulants and alcohol use.

In the EPP, CU, even at low dose (50), has been associated with violence (58) and severe violence (52). Some studies have shown that this association was not found for other drugs (51, 52), and could be potentiated by the precocity of the consumption (59). It seems also that dose-response (39, 46, 60, 61), trajectory of consumption (56), and persistence of CU (39) could be important variables in the association between CU and VB. In studies conducted in prisoners (53) and in the general population (61), it has been shown that the average age at which the violent patients started using cannabis was 15 years [two years earlier than the non-violent patient group (62)], and that the precocity of CU was a risk factor for VB (62) .

Despite recent evidence on the link between CU and VB in psychosis, the relationship needs to be further explored, as there are still contradictory results, some studies not finding a link between CU and VB (38, 63). These contradictory results may be due to theoretical limitations, such as differing definitions and assessments of violence; or methodological limitations, such as: the predominance of cross-sectional or retrospective studies, the failure to account for confounding factors (i.e., previous delinquent behavior, co-morbid personality disorders, dose of used substance, etc.), the lack of longitudinal studies with repeated assessments over time and on short follow-up periods, which are necessary to assess the short and long term effects of CU, and the direction or bi-direction of the associations. On this matter, two recent studies (56, 57) support unidirectionality but need to be confirmed by others. It should also be noted the scarcity of data on offender samples, or lack of objective and detailed data on the CU characteristics such as type, dose, and frequency of use (38). Moreover, there are insufficient empirically data of what underlies this relationship (38, 39, 63).

## Explanatory Hypotheses of the Link Between Cannabis Use and Violent Behavior

Several hypotheses have been proposed to understand the link between CU and VB. A first involves *pharmacological explanations*, with CU chemically dysregulating emotion processes, which could contribute to the development of VB (40, 46, 61, 64, 65). This mechanism has also been related to *the type, amount*, and *frequency* of CU (38), or withdrawal symptoms (63). A second hypothesis suggests that CU may predispose to VB (61, 66, 67) through the induction of *depersonalization and feeling of persecution*. A third hypothesis suggests that *pre-existing adolescent conduct disorders* (12, 68) or predisposition for aggression (54) could also lead to early CU and VB. For example, Rioux et al. (59) have shown that delinquency at age 12 and associations with deviant peers is associated to early onset of CU, which in turn predicts violence. Other hypotheses suggest that the *lifestyle* of individuals and some *interpersonal and contextual factors* (69), such as subcultures, may encourage both violence and drug abuse (69). Or, that the interaction with a delinquent environment, may predispose to CU, and, in turn, CU may contribute to delinquency and illicit trafficking (69), suggesting a complex and reciprocal relationship between these factors (61). In the deviance theory, violence and CU are both considered as deviant behaviors in the context of a “*general deviance syndrome*.” Violence and CU would in this case not be causally linked, but rather associated to a common dimension, such as antisocial personality disorder. In this line, biopsychosocial hypotheses (69) suggest that a combination of interactions between distal (for example, family history of substance use) and proximal biopsychosocial factors (for example, emotional arousal) would constitute the pathways by which CU may be related to violence.

In addition to these elements, and if we focus specifically on psychosis, CU may also be associated with other dimensions that have all been related to VB (34, 37), and which can mediate this relationship. These include reduced insight and adherence to treatment (40, 52, 70–72), increased anxiety, anger, hostile attribution bias, suspiciousness, and positive symptoms (40, 73). CU may exacerbate these dimensions, and particularly positive psychotic symptoms, which in turn may increase VB (46, 64, 74). One dimension frequently mentioned as mediating this relationship is impulsivity (35, 40, 61, 75). In line with some literature data (50, 76) and the results of our own research, we hypothesize that the latter factor, impulsivity, may be a mediating the effect of CU on VB. We argue that impulsivity (41) could be an intermediate variable in the relationship between CU and VB, simultaneously inducing consumption through its inhibitory defect (50, 76), and be impacted by consumption in a self-feeding mechanism (77).

## Role of Impulsivity in the Relationship Between Cannabis Use and Violent Behavior in Psychosis

### Impulsivity and Violent Behavior in Psychosis

Initially studied in offenders with personality disorders, impulsivity has recently been the focus of work in psychosis, showing its importance in this pathology (78–83). A meta-regression analysis of risk factors for VB in psychosis (EPP or chronic patients) found that, among several dynamic psychopathological risk factors, high impulsivity was an important one (34, 35). Several studies have shown the impact of impulsivity on VB with different populations [chronic patients (34–36, 84, 85), EEP patients (37, 50, 86), and forensic populations (85)] and different measures of impulsivity (clinical self-report or hetero-report and behavioral scales) (73, 86–88). In the EEP, prospective cohort studies have also shown that impulsivity is associated with serious aggression (37, 50), either directly or indirectly. Several authors suggest that the relationship between impulsivity and VB may be mostly indirect (89) and mediated by other factors, like early conduct disorder, antisocial personality disorder (81), psychopathic trait, affective instability and negative emotions (73), as well as positive symptoms (78, 79). In addition, substance use is frequently highlighted as a dimension that may also underlie this link (78, 81, 90).

### Impulsivity and Cannabis Use in Psychosis

#### Clinical Domain

In the general population, it has been shown that impulsivity can be associated with substance use, both in its early onset (59) and in its persistence over time (91). Impulsivity is one of the most important factors underlying CU, and impulsivity-related traits may be markers and predictors of substance use (92). It has also been suggested that impulsivity may increase susceptibility to drug or facilitate an “uncontrolled response” to drug use (93). A recent review on impulsivity in a forensic population showed that impulsivity could be simultaneously a determinant and a consequence of substance abuse (77, 94).

In psychosis, it is not yet known whether it is the pre-existing impulsive trait that drives the use or whether it is the use that increases impulsivity (76, 78, 95). Various authors show that patients who use cannabis are more impulsive (76, 79, 96) than those who do not. Repeated CU may also increase impulsivity (97–100) and impulsivity is thought to play a key role in the development and maintenance of drug dependence, including cannabis (70, 78, 98, 101–103).

In a longitudinal cohort, and after controlling for various other dynamic factors (other substances and psychopathological factors), we found that CU is one of the two main risk factors associated with severe violence in the EPP. It has an impact on VB independently, but also in association with impulsivity (52). A second study analyzed the relationship between CU, its precocity, and impulsivity levels over the course of a three-year treatment program, in violent and non-violent groups of patients. The results showed (75) that there was a significant increase of impulsivity during treatment in the violent patient group whose patients are early cannabis users, i.e., who started CU before age 15. This is to be contrasted with results of the violent patients who started CU after 15 years of age, and non-violent patients, whose impulsivity levels remained stable during the program.

#### Neurobiological Domain

Over the past decade, research has shown increased interest in identifying neurobiological factors, such as genetic influences, neurotransmission, or brain structures and circuitry, which may explain the association between drug use and impulsivity.

Neurobiological research on neurotransmitters examined levels of glutamate, GABA, dopamine, serotonin, and norepinephrine in brain structures associated with impulsivity and drug use as potential factors explaining the relationship between impulsivity and CU (104). This research has shown that fluctuations in certain levels of neurotransmitters can alter reward control and may be critical for drug compulsion (104, 105). For instance, glutamate levels in the dorsal ACC have been associated with delay discounting in drug users, leading to the idea that regulation of glutamate levels may alter the reward processing and thus minimize drug reinforcement. In addition, norepinephrine has also been associated with impulsive behavior and addictions because of its impact on the reward effects of addictive behavior (106). In a clinical study, regulation of norepinephrine levels by adrenergic modulators was reported to have an effect on cessation of drug use, by reducing pivotal elements such as stress-induced craving and the effect of drug alerts (107). Finally, serotonin levels have been linked to drug use and impulsivity.

Neurobiological research on the genetic side has identified CNR1 as a promising candidate to explain the relationship between CU and impulsivity. CNR1 is a cannabinoid receptor-related gene that has been linked to CU (108). Interestingly, one study examined whether impulsivity and CU related problems can be explained by CNR1 genes. Specifically, CNR1 variation appears to moderate the relationship between the degree of impulsivity and CU related problems (e.g., social relationships, self-esteem, motivation and productivity, as well as work and finances). Moreover, CU with higher levels of impulsivity associated with CNR1 risk variants showed a higher risk of developing CU related problems. Although this is only one of the findings of genetic research attempting to explain the association between CU and impulsivity, and that many other genes may also be promising candidates, such as CADM2 or FAAH (109), this provides an example of the potential of studying genes to explain the association between impulsivity and CU.

Further research focused on the link between CU and the structures and functions of brain areas related to action control (110, 111). Research that focused on the link between CU and impulsivity-related brain structures reported structural alterations, such as decreased cortical volume, in cannabis users (112–116). Structural abnormalities were also reported in patients suffering from schizophrenia, in the EPP (117–122), chronic psychotic patients (123), as well as in subjects at high risk for developing psychosis (124, 125). In these populations, the literature suggests that, due to genetic factors, the prefrontal areas (OFC, Dorso-Lateral Pre-frontal Cortex, DLPFC, and Anterior Cingulate Cortex, ACC) and the cerebellum, which have also been reported to play a critical role in impulsive behavior (126–130), appeared to be extremely sensitive to CU. Furthermore, there is evidence that this may be particularly the case in early life CU (131). The authors explain that, in contrast to adulthood, adolescence is a period of neuromaturation, which renders the brain particularly vulnerable to the effects of drugs (132).

Finally, some research tried to model impulsive behavior through fronto-striatal circuits. This research reported that impulsive individuals have a more active fronto-striatal network than non-impulsive individuals (133–137). Importantly, this has been reported in both patients with psychosis and healthy participants. The fronto-striatal circuits that are involved in action control include fibers that project from the prefrontal cortex areas through the basal ganglia and end in the motor cortex. Aron and Poldrack (138) suggested three fronto-striatal networks, each with a specific role in action control: the hyperdirect pathway (stop of programmed action), the direct pathway (action initiation) and the indirect pathway (action termination). The hyperdirect pathway—due to its role in the suppression of any programmed action—appears to be of major interest. Indeed, a dysfunction of this pathway should lead to difficulties in controlling action suppression and thus result in impulsive behaviors. More relevantly, some studies reported that drug use was related to fronto-striatal circuit alterations (139–141), giving rise to the hypothesis that cannabis and impulsivity may be linked through the effect of cannabis on such circuits.

## Discussion

Overall, the hypothesis of an involvement of impulsivity in the relationship between CU and VB seems probable and should be further explored at the neurobiological and clinical levels. Neurobiological research has shown how genes, neurotransmitters, gray matter volume, and brain circuitry may be behind the link between cannabis and impulsivity. Three results seem particularly insightful. First abnormal concentrations of neurotransmitters in certain areas seem to play a crucial role in impulsivity, triggering difficulties in discounting delay, which plays a critical role in compulsive drug use. Second, research on brain areas and circuits associated with impulsivity suggests that drug use is associated with different levels of brain volume. This appears to be particularly pronounced in schizophrenia and in early drug users. Third, better specification of fronto-striatal circuits indicated relation to impulsivity, which could be particularly relevant to understand the link between impulsivity and CU.

While these are promising research findings, most of them focused on general drug use and were not specific to CU. In this vein, most of this research focused on the relationship between drug compulsive process, neurobiological factors and impulsivity leading. Although we might concede that drug compulsion should be a common process in alcohol, cocaine, and cannabis use, it would be important to focus on the specific neurobiological factors behind the relationship between CU and impulsivity. Moreover, the direction of the association between CU, impulsivity, and neurological factors is not yet clear. Researchers suggest that long-term consumption alters the structures and functions of frontal areas known to play a role in impulsive behavior, such as the motor inhibition region (110). This in turn would lead to increased levels of impulsivity. Other research on neurotransmitters or genes suggests that neurobiological changes in neurotransmitters or genes responsible for impulsivity may confer a higher risk for drug use. According to this view, the link between impulsivity and CU would be the result of an increased risk of CU in individuals who are more impulsive due to neurobiological factors.

Consequently, further research is needed to understand how CU and impulsivity are linked through neurobiological factors. Future research should focus on the longitudinal aspect of CU and neurobiological components. In particular, it would be important to examine the development of impulsivity—related brain areas or circuits, for instance, and the scores of impulsivity in users and non-users over time. Moreover, research should separate schizophrenic and non-schizophrenic patients, as studies show that CU and brain structures related to impulsivity are closely related in patients suffering from schizophrenia.

At clinical level, the hypothesis of an involvement of impulsivity in the relationship between CU and VB should also be explored in more depth. This should be done on the basis of longitudinal studies (too scarce) and by monitoring the evolution of impulsivity and consumption (with repeated assessments). The interactions between these two dimensions and with VB should be assessed, as well as the directionality or bi-directionality of these associations [which remains debated in studies targeting young adults (54)]. Longitudinal studies should take into account possible confounding or mediating factors (mentioned above in the sections on the links between VB and CU), on the one hand, and VB and impulsivity, on the other hand. In addition to violence-specific factors, a recent review (142) of longitudinal studies investigating the impact of CU on global outcomes in psychosis, recommends that factors such as the type of psychosocial intervention received by the patients, medication adherence, and the type of antipsychotic medication prescribed should be taken into account.

The study of the involvement of impulsivity in the relationship between CU and CV should also take into account the different subgroups of violent patients (81, 143). Within the two main subgroups identified in studies (81, 143), i.e., patients with or without early conduct disorders, impulsivity and CU could play a role, but in different ways, in the processes leading to VB. The subgroup with early conduct disorders (including antisocial behavior and substance use) may involve these factors early in the life course of patients and in a complex and reciprocal way. More, in this profile, impulsivity and substance use may also reflect (or be associated with) co-morbid personality disorders, especially anti-social ones (13, 144). In the subgroup of patients without early conduct disorder, who display VB at the time of onset of psychotic disorders, the link between CU, impulsivity and VB could be exacerbated by psychotic symptoms (73). Conversely, CU may exacerbate impulsivity and symptoms, which in turn increase the risk of VB.

Studies have shown that patients respond differently to treatment depending on their profile (143, 145). So, early intervention programs in psychosis could implement an identification of patients with early conduct disorders, high impulsivity and CU, as well as trace the onset of CU to identify early and persistent users. The use of tools to assess risk factors for VB [such as the HCR20 (146, 147)], CU and impulsivity, could be useful. Recent studies have shown the feasibility and value of these tools into mental health services. This could contribute to increase the preventive aspect of such programs.

In order to reduce VB, recent literature has shown the interest and relevance of targeting the factors associated with these behaviors, in addition to interventions targeting psychotic disorders (148). The “Integrated treatment” (149) [for example including motivational approach, cognitive-behavioral methods, or group counseling (150)] focuses on both substance use and psychosis and has been shown to be effective. Assessment of the level of impulsivity and subsequent interventions to reduce it [e.g., with cognitive-behavioral therapies, cognitive remediation, management of stressful situations as well as some anti-psychotic treatments (73, 150)] would also be necessary. However, there are not yet longitudinal studies, conducted with strict randomized controlled trials, demonstrating the effects of these interventions on impulsivity levels in this population.

Examining these issues is of primary importance, not only to reduce VB but also to ensure better psychopathological outcomes for patients, better care outcomes, and better insertion into society.

## Author Contributions

VM wrote the manuscript with the contribution of DF. ED-G revised the manuscript. All authors contributed to the article and approved the submitted version.

## Conflict of Interest

The authors declare that the research was conducted in the absence of any commercial or financial relationships that could be construed as a potential conflict of interest.

## Publisher's Note

All claims expressed in this article are solely those of the authors and do not necessarily represent those of their affiliated organizations, or those of the publisher, the editors and the reviewers. Any product that may be evaluated in this article, or claim that may be made by its manufacturer, is not guaranteed or endorsed by the publisher.
